# Role of mismatch repair in aging

**DOI:** 10.7150/ijbs.64953

**Published:** 2021-09-21

**Authors:** Jie Wen, Yangyang Wang, Minghao Yuan, Zhenting Huang, Qian Zou, Yinshuang Pu, Bin Zhao, Zhiyou Cai

**Affiliations:** 1 Chongqing Key Laboratory of Neurodegenerative Diseases, Chongqing, 400013, China.; 2 Department of Neurology, Chongqing General Hospital, University of Chinese Academy of Sciences, Chongqing, 400013, China.; 3 Department and Institute of Neurology, Guangdong Medical University, Guangdong, 524001, China.; 4 Guangdong Key Laboratory of aging related cardio cerebral diseases, Guangdong, 524001, China.

**Keywords:** aging, mismatch repair, genomic instability, telomere attrition, epigenetic alterations, mitochondrial dysfunction

## Abstract

A common feature of aging is the accumulation of genetic damage throughout life. DNA damage can lead to genomic instability. Many diseases associated with premature aging are a result of increased accumulation of DNA damage. In order to minimize these damages, organisms have evolved a complex network of DNA repair mechanisms, including mismatch repair (MMR). In this review, we detail the effects of MMR on genomic instability and its role in aging emphasizing on the association between MMR and the other hallmarks of aging, serving to drive or amplify these mechanisms. These hallmarks include telomere attrition, epigenetic alterations, mitochondrial dysfunction, altered nutrient sensing and cell senescence. The close relationship between MMR and these markers may provide prevention and treatment strategies, to reduce the incidence of age-related diseases and promote the healthy aging of human beings.

## Introduction

The proportion of people over 65 is rapidly increasing in most industrialized countries 1. This demographic milestone will be accompanied by a significant increase in age-related illnesses such as Alzheimer's disease, cardiovascular disease, and cancer 2. Aging is a progressive degenerative state, accompanied by tissue stem cell depletion, tissue inflammation, matrix changes, cell senescence, and metabolic dysfunction. The current status of aging research has many similarities with cancer research. Cancer and aging share a common origin and can be thought of as two different manifestations of the same underlying process, which is the accumulation of cell damage 3. In addition, many diseases of premature aging, such as Werner syndrome and Bloom syndrome, are the result of increased accumulation of DNA damage 4.

The integrity and stability of DNA are constantly challenged by exogenous physical, chemical, and biological media, as well as endogenous threats, such as DNA replication errors, spontaneous hydrolysis and reactive oxygen species (ROS). Meanwhile, the completeness of genetic information depends to a large extent on the accuracy of the DNA replication process 5. In order to maintain the integrity of the genome, cells have a variety of mechanisms for repairing DNA damage, including MMR, base excision repair (BER), nucleotide excision repair (NER), homologous recombination (HR), non-homologous terminal connection (NHEJ) 6, 7. This article reviews the close relationship between MMR and aging mechanism from several aspects, in order to provide some new insights into how to fight aging and prevent aging-related diseases.

## MMR system

**Figure 1:**
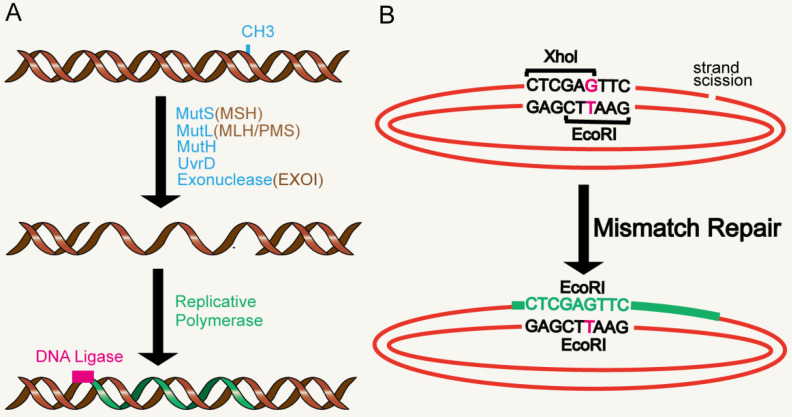
** MMR reaction. A.** MMR excision-resynthesis process. The reaction degrades one strand and uses the complementary strand as a repair template to eliminate the mismatch. The γ-proteobacteria components directing the specific excission are shown in blue. Bacterial (besides γ-proteobacteria), archaeal, and eukaryotic components are shown in brown. Resynthesis at the exonuclease gap is accomplished by replicating polymerase, and the remaining strand breaks are blocked by DNA ligase. **B.** Illustration of a simple MMR DNA substrate that contains overlapping restriction sites containing mismatches leading to restriction resistance to endonuclease.

The DNA MMR system is ubiquitous among organisms, from prokaryotes to eukaryotes. In 1964, MMR was conceived simultaneously by Robin Holliday explaining genetic transformation in yeast and by Evelyn Witkin explaining brominated nucleotide processing in bacteria 8, 9. MMR, an evolutionarily conserved process, corrects the mismatches that occur during DNA replication and escape proofread 10. Its primary function includes excision-repair, through which a region containing the wrong base in newly synthesized DNA chain is removed and resynthesized 11 (Figure 1A). The main proteins involved in the MMR system are MLH1, MutS protein homology 2 (MSH2), MutS homology 6 (MS6) and PMS1 homology 2 (PMS2), which together interact in the form of heterodimers, whereby MSH2 binds to MSH6 or MSH3 to form MutSα and MutSβ complexes respectively, while MLH1 binds to PMS2 or MLH3 12-14. The basic MMR reaction is most easily understood in Escherichia coli. MMR is initiated by MutS homodimer upon recognition of mismatches, which then recruits MutL (also a homodimer complex) to the mismatch sites. MutL interacts with the third MMR component called MutH, resulting in the activation of the potential endonuclease activity of MutH and formation of a gap in the newly replicated chain. Chain recognition is a basic feature of MMR, which limits the use of amphiphilic chains as templates for DNA re-synthesis, thus eliminating misincorporation errors 11, 15, 16. Eukaryotic MMR is similar to bacterial MMR in mechanism of action. However, its mismatch recognition uses two MutS homologous complexes, MSH2-MSH6 and MSH2-MSH3, which have partially overlapping mismatch recognition specificity, rather than a single MutS homodimer as in prokaryotes. The third MSH complex (MSH4-MSH5) does not have MMR-related functions, yet is very important in the process of meiosis and recombination 17, 18. Reconstruction of the MMR reaction in E. coli began in 1983 using DNA substrates containing two overlapping restriction enzyme sites with central mismatches 19 (Figure 1B). The most significant MutL activity in human cells is provided by MutLα heterodimer MLH1/PMS2. In addition, human MLH1 also exists as heterodimers containing PMS1 and MLH3, called MutLβ and MutLγ 20, 21. Studies have found that MMR plays an important role in the process of aging.

Genomic instability, one of the key hallmarks of aging, leads to stem cell exhaustion and triggers inflammation. Genomic stability is supported by complex repair mechanisms, damage tolerance, and checkpoint pathways that counteract DNA damage. DNA damage is responsible for the development of cancer and many age-related diseases 22. Causal evidence for a link between lifelong increase in genomic damage and aging comes from studies on mice and humans, showing that defects in DNA repair mechanisms cause accelerated aging in mice and are the basis for several human genetic syndromes, including Werner syndrome, Bloom syndrome, xeroderma pigmentosa, and trichothiodystrophy 22-24. Several mutation patterns in somatic cells are either associated with exposure to substances that damage DNA or with genomic instability due to DNA repair failure 25. Loss of MMR function induces a hypermutant phenotype clinically identified as microsatellite instability (MSI). dMMR (MMR *deficiency*)/MSI tumors exhibit proteome-wide protein instability associated with numerous unstable mutations 26. Summarizing the specific relationship between MMR and the aging process may provide some strategies for humans to delay aging.

## MMR and telomere attrition

**Figure 2:**
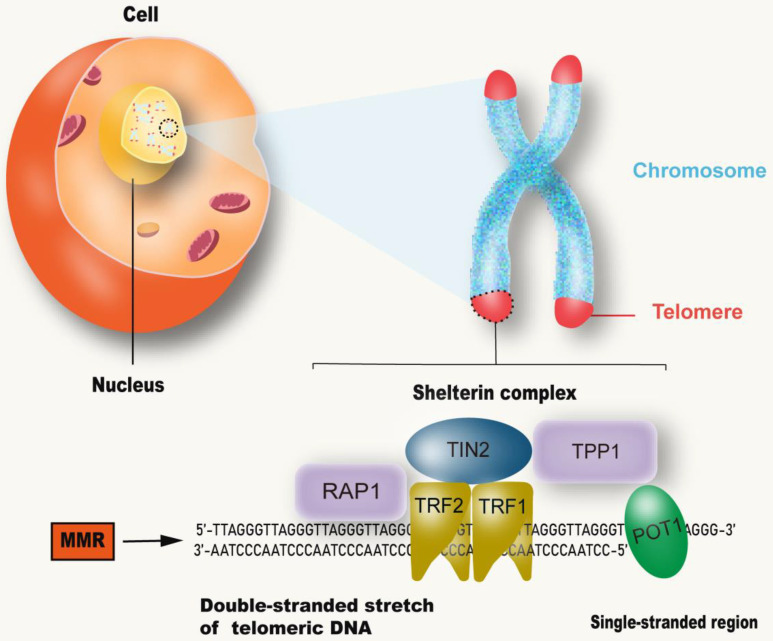
** MMR and telomere attrition.** Telomeres are located at the end of linear chromosomes in eukaryotic cells and are protected by polymers composed of six protein subunits: TRF1, TRF2, TPP1, POT1, TIN2 and RAP1. The absence of MMR system can accelerate the shortening of telomeres.

Although the process and potential molecular mechanisms of aging have not been fully understood 27, more evidence points to telomeres, which are believed to be the initiators or amplifiers of the molecular circuits that drive the aging process and related diseases 28 (Figure 2). Telomere shortening appears to be one of the biological manifestations of aging 3. Short telomeres, rather than average-length telomeres, determine cell viability and chromosome stability 29. Reaching the critical telomere length can lead to replicative senescence or programmed cell death 30. Telomeres are made up of repeated nucleotide sequences to form a “cap structure” which function to maintain the integrity of chromosomes. Human telomere maintenance related gene defects are associated with reproductive and somatic degenerative diseases, such as congenital keratosis, idiopathic pulmonary fibrosis, ulcerative colitis, etc. 31. Telomeres are strictly regulated by telomerase. In most eukaryotes, telomerase uses a complete RNA template consiting of telomerase RNA and telomerase reverse transcriptase (TERT), to synthesize telomere repeat sequences (TTAGGG) at the end of chromosomes 32. These repeat sequences compensate for telomere loss caused by incomplete replication at the end of the genome 33. In humans, telomerase expression is up-regulated during embryonic development and cancer, and mutations that damage telomerase function can lead to disease 34. Furthermore, telomeres are covered by special proteins called protegerin complexes, which are polymers composed of six protein subunits: TRF1, TRF2, TPP1, POT1, TIN2 and RAP1 35. Protegerin subunits organize telomeres into spherical structures through complex interactions with telomere DNA 36, 37. These advanced structures of telomeres suppresses DNA damage signals from telomere terminals, prevent DNA repair mechanisms from fusing the terminals through recombination or classical/alternative NHEJ, and regulate telomerase pathway and activity. Accordingly, mutations in the above components can destroy the protegerin-telomere complex, leading to terminal fusion and premature senescence 38, 39.

For example, defects in the MMR system were suggested to lead to HR of telomere ends resulting in telomerase-independent telomeres in gastric cancer 40. The DNA MMR system maintains the stability of the genome not only by repairing DNA replication errors, but also by preventing homologous chromosome recombination 41. Indeed, the absence of MMR helps cells overcome the cellular crisis caused by telomere dysfunction through telomere recombination in telomerase-deficient yeast or mammalian cells. The loss of MMR function promoted the proliferation of telomerase deficient yeast cells 42, inhibited telomerase activity and accelerated the ALT-like telomere elongation of MMR-deficient human colon cancer cells 43. DNA MMR is essential for genomic stability and inheritance of MMR genes. Mutated MMR genes are most commonly caused by MSH2 or MLH1, leading to cancer susceptibilities such as in Lynch syndrome and hereditary nonpolyposis colorectal cancer (HNPCC). The average telomere shortening rate of MSH2 deletion clones was significantly higher than that of control clones. This was the first evidence that MSH2 deficiency alone can lead to accelerated telomere shortening in normal human cells 44.

Pathological telomere shortening can lead to genomic instability and lymphatic transformation. At least two main pathways have been proposed to lead to colorectal cancer (CRC): loss of heterozygosity caused by chromosome instability and DNA MMR defects caused by microsatellite instability 45-47. Severe genomic instability in telomere crisis accelerates secondary genetic changes that lead to carcinogenesis and thus emphasize the significance of pathological telomere length changes in cancer pathways, including colorectal cancer 48-50. Relative telomere length (RTL) is closely related to clinicopathological predictive markers of sporadic colorectal cancer. The expression of MMR proteins including MLH1, MSH2, PMS2, MSH6 and p53 protein is normal in sporadic colorectal cancer 51. In the late stage of tumorigenesis, telomere loss leads to genomic instability and telomerase activation promotes immortalization 52.

## MMR and mitochondrial dysfunction

**Figure 3:**
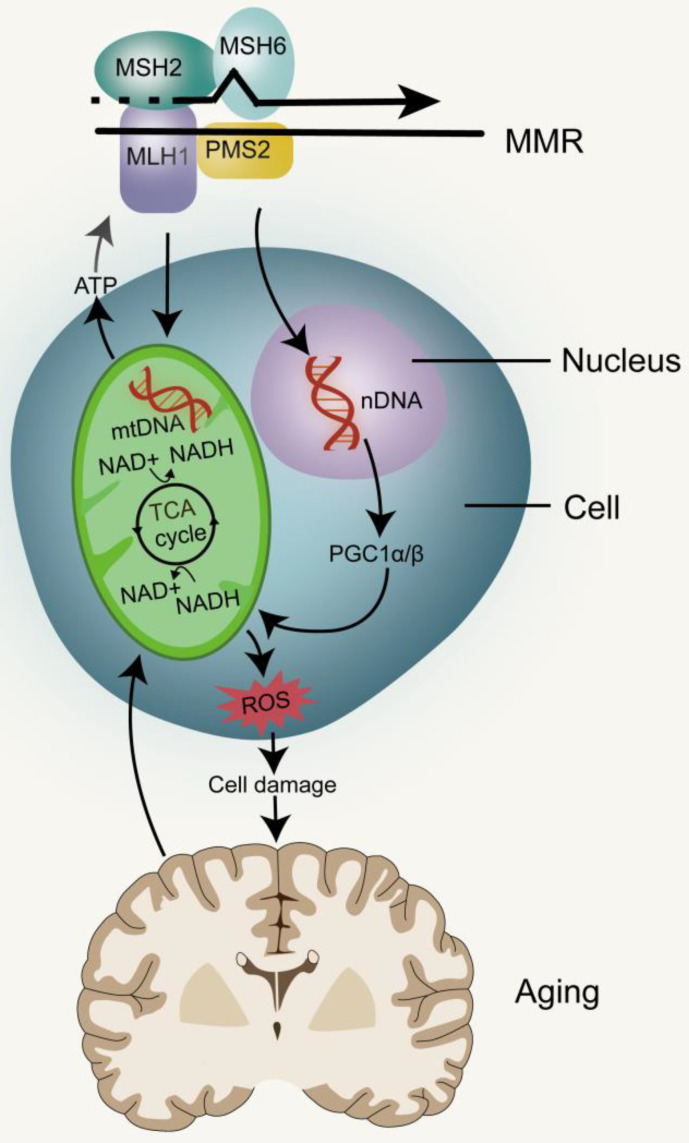
**MMR and mitochondrial dysfunction**. The stable inheritance of mtDNA and nDNA requires the participation of MMR. mtDNA mutations directly promote mitochondrial dysfunction. nDNA damage can lead to mitochondrial dysfunction by inhibiting PGC1α and PGC1β, and eventually lead to aging. Impairment of mitochondrial function can affect the production of ATP thereby reducing the energy needed by MMR, as well as produce metabolic toxins such as ROS to promote aging.

Mitochondria are important organelles for executing and coordinating various metabolic processes in cells and participating in ion homeostasis, cell growth, redox state, and cell signaling (Figure 3). They are also main source of intracellular ATP and play prominent roles in cell life and death 53, 54. Mitochondria are unique because they contain their own DNA (mtDNA), which encodes many proteins that are essential for the assembly and activity of mitochondrial respiratory complexes, promote mitochondrial replication, transcription and repair of mtDNA. With a few exceptions, animal mitochondrial DNA encodes 13 key proteins, and about 1500 nuclear DNA (nDNA)-encoded proteins are destined for the mitochondria 55. Defects or mutations in mitochondrial DNA can lead to several diseases, including cancer, and mitochondrial diseases, while damaged mtDNA can be eliminated by mitosis 56-58. The mutation rate of mtDNA is significantly higher than that of nDNA, because it is close to high concentrations of toxic metabolites and the repair mechanism is relatively inefficient 59. DNA deletion and point substitution can lead to serious disorders of electron transport chain (ETC) function, mitochondrial genome replication and mitochondrial gene expression 7. ATP is produced through the Krebs cycle in the mitochondrial matrix and oxidative phosphorylation (OXPHOS) in the mitochondrial inner membrane, with ROS and heat as byproducts 55. Mitochondrial dysfunction leads to reduction of ATP produced oxidative phosphorylation, inability to regulate excessive ROS and nitrogen production, calcium regulation disorders, opening of permeability transition pores and initiation of apoptosis 60. Studies in the free-living transparent nematode, *Caenorhabditis elegans*, have shown that reducing the activity of mitochondrial ETC in the early stage of life leads to extensive chromatin recombination. This is often required to activate mitochondrial unfolded proteins in response to stress, a process that promotes the restoration of mitochondrial protein homeostasis and regulate stress-induced lifespan 61, 62. Therefore, partial inhibition of mitochondrial activity has been shown to prolong the lifespan of worms, flies and mice, but complete or nearly complete loss of mitochondrial function is harmful to the organism 63-66. The causes of mitochondrial dysfunction include changes in mitochondrial dynamics caused by the imbalance between fission and fusion events, quality control of defects caused by mitosis, a form of bulk autophagy peculiar to organelles and protein degradation targeted at defective mitochondria 67. mtDNA mutations, deletions or impaired DNA replication are the most common causes of mitochondrial dysfunction 68.

The general mechanism of MMR includes locating the mismatch and repairing the newly synthesized chain through excision and DNA re-synthesis, followed by reconnecting to complete the repair process 69. Spontaneous errors in the mechanism of mtDNA replication can lead to point mutations and deletions. The MMR system corrects post-replication errors and maintains genetic stability. Efficient MMR systems also identify and cut sequence variants in mtDNA 70. For a long time, research work was focused on the identification of MMR proteins located in the nucleus, because few key factors involved in MMR had been identified in mitochondria, and only relative recognition complexes MutSα and MutSβ had been detected in mitochondrial MMR activity. In fact, the mtDNA repair depends to a large extent on the mechanism of nDNA repair 69. YB-1 protein is another nuclear MMR factor, which is partially located in mitochondria. When small interference RNA (siRNA) was used to inhibit the expression of Yb-1, the activity of MMR in mitochondrial extract decreased by more than 3 times 71. So far, no other participants of nuclear MMR have been detected in mitochondria 7. mtDNA has a large non-coding sequence, the displacement-loop (D-loop), which contains the essential elements for transcription and replication 72, and studies have shown that this region suffers more damage than other regions of the mtDNA 73. As the MMR system was damaged, sequence mutations in the D-loop region were not repaired, resulting in impaired mtDNA transcription. It has been shown that in diabetes, mitochondrial dysfunction in the retina and capillary cells cause initiation of apoptosis, mtDNA damage, electron transport chain damage, mtDNA biogenesis, and copy number reduction 74, 75. Mitochondria become dysfunctional, and even after hyperglycemic condition is reversed to normal, they remain dysfunctional, leading to the development and progression of diabetic retinopathy. Therefore, strategies that target mtMMR mechanisms can help maintain mitochondrial homeostasis 76. MLH1, one of the key proteins involved in the MMR pathway, suppresses a few mitochondrial genes, including POLG and PINK1, that induce synthetic death in MLH1-deficient cells 76-78. Recent studies have shown that the loss of MLH1 is related to mitochondrial metabolism disorders, with basal oxygen consumption rate reduced and spare respiratory capacity reduced. In addition, loss of MLH1 resulted in decreased OCR, decreased complexⅠactivity, and increased reactive oxygen species 79. The relationship between mtDNA and base mismatch has been doubted for a long time, and better understanding of its intricacies is still an important challenge in aging research.

Elevated levels of DNA damage activate p53, and the increase in p53 levels results in mitochondrial dysfunction by inhibiting PPARγ coactivator1α (PGC1α) and PGC1β (which promotes mitochondrial biogenesis). The p53-mediated mitochondrial dysfunction triggers the DNA damage cycle by affecting the production of ROS, Fe-S clusters and NADH/NAD, which leads to further activation of p53 and mitochondrial damage 80. The deficiency of ATP production and the increase of ROS level can promote cell senescence. With the aging of cells and organisms, mitochondria gradually become inefficient and potentially toxic; the efficacy of the respiratory chain tends to weaken, thereby increasing electron leakage and reducing the production of ATP 81. The initiation of MMR in eukaryotes depends on the recognition of DNA mispairs by partially redundant MSH2-MSH6 and MSH2-MSH3 heterodimers which are homologues of bacterial MutS homodimers 82, 83. MSH2-MSH6, MSH2-MSH3 and MutS belong to the ABC family of ATPases, and there are two nucleotide binding sites at the interface of their dimers. When the MSH2-MSH6 complex binds to mispairs, it also binds to ATP at its two nucleotide binding sites, resulting in a conformational change. This ATP-activated MutS state promotes its interaction with one or more MutL proteins. ATP binding, hydrolysis and ADP release are intrinsically related to the conformational changes that play a key role in MMR 17, 84-86. MMR of DNA can lead to mtDNA mutation and nDNA damage, which in turn leads to mitochondrial dysfunction and accelerates the aging process. The key potential factor of cell change in the process of aging is metabolic disorder and mitochondrial dysfunction in turn hinders metabolic processes, resulting in a vicious circle 87.

## MMR and epigenetic alterations

Epigenetics, which includes DNA methylation, histone modification, chromatin remodeling and non-coding RNA, represents a reversible genetic mechanism that occurs without changing the underlying DNA sequence 3, 88, 89. Although all cells have the same DNA, the epigenetic mechanism determines the fate of cells, such as whether they become hepatocytes or neurons, as well as the maturation of different types of cells. Importantly, epigenetics can be influenced by environmental factors. Thus epigenetic alterations can be spontaneous or driven by external or internal factors. For example, identical twins share the same DNA, and epigenetic markers are similar among young twins. Studies on human longevity have shown that genetic factors can explain a small portion of the observed differences in the lifespan of identical twins (20% to 30%), while the bulk of remaining variations are thought to be caused by epigenetic drift over their lifetime 90-93. Epigenetic alterations are thus related to aging, however, how this affects aging is not clear. Studies have suggested increased histone H4K16 acetylation, H4K20 trimethylation or H3K4 trimethylation, as well as decreased H3K9 methylation or H3K27 trimethylation, as age-related epigenetic markers 94, 95. The MMR pathway initiates epigenetic alterations during inflammation-induced tumorigenesis 96.

**Figure 4:**
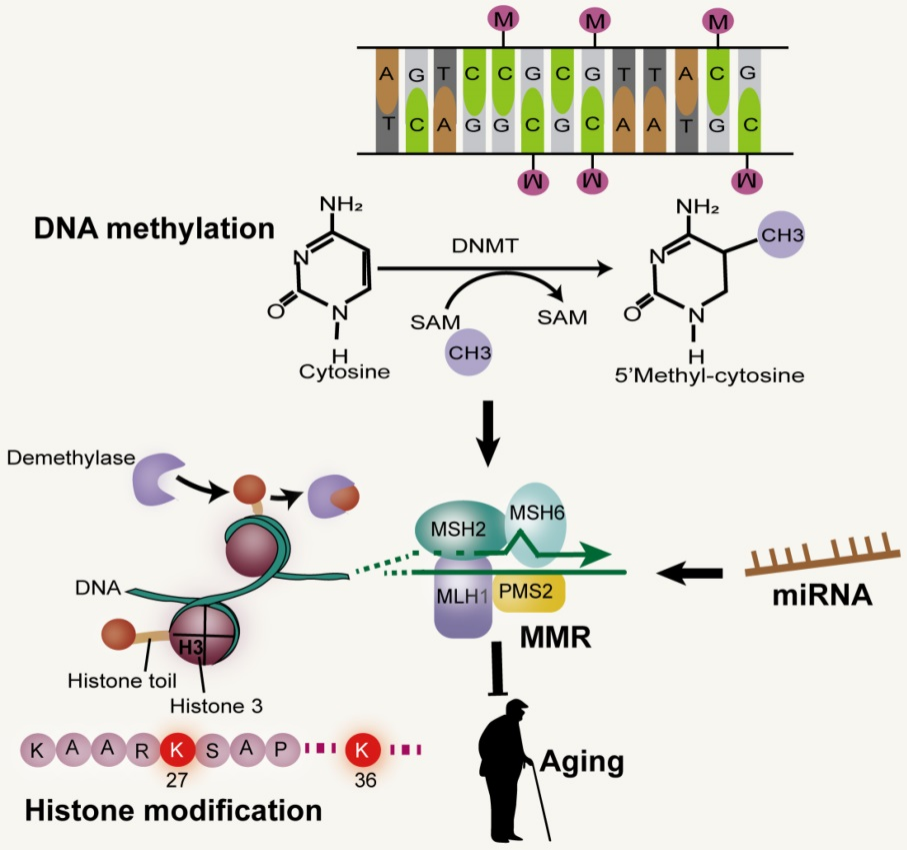
** MMR and epigenetic alterations.** Mammalian MMR is related to Dnmt1 and CpG. Reductions of H3K36me3 levels can impair the MMR pathway. miRNA, a non-coding RNA, has been shown to be involved in the DNA MMR.

DNA methylation at C of CpG dyads (CpG) in vertebrate genomes is vital for gene regulation, genome stability and development 97. In the early stages of development, CpG methylation increases in the brain. Although there is no significant change in the global level during aging, the CpG methylation pattern seems to be changed. In some tissues, reduced CpG methylation was observed in repetitive sequences, including transposable elements 98. The high fidelity of DNA methylation patterns in mammals after each cell division is regulated by DNA methyltransferase (Dnmt) 94. Studies have shown that the normal function of post-replicative DNA MMR in mammalian cells depends on the existence of genomic CpG and the maintenance of Dnmt1, independent of its catalytic activity. Furthermore, the efficient monitoring of mammalian MMR is achieved through the hemi-CpG-Np95 (Uhrf1)-Dnmt1 axis, through which the MMR monitoring surveillance complex is recruited by Dnmt1 to the replicated DNA, a process that requires it to bind to MutSα and Np95 on the hemi-methylated CpG site. Therefore, the efficiency of MMR monitoring of mammalian genome *in vivo* is improved at the epigenetic level 97 (Figure 4). In addition, MSH2 and MSH6 recruits epigenetic silencing proteins Dnmt1 and EZH2 to oxidative damage sites through a protein-protein interaction-dependent mechanism. It has been proven that the heterodimer of MMR protein, MSH2-MSH6, is involved in the recruitment of Dnmt1 to chromatin impaired by oxidative damage. The role of Dnmt1 in the oxidative damage site is to reduce transcription and potentially block the repair process by transcriptional interference 99.

Studies have shown that the level of H3K27me3 in wild-type fruit flies increases with age. One possible reason that aging promotes the drift of H3K27me3 modification is that age-related DNA damage reduces the fidelity of epigenetic markers. Methylation of histone H3K9 and H3K27, acetylation of histone H3 and citrullination of histone H2A1R3 were up-regulated in patients with colorectal cancer 100. UV-C radiation can induce the increase of H3K27me3 mediated by polycomb repressive complex2 (PRC2) in the silkworm, Bombyx mori 101. Meanwhile, in Neurospora crassa, the increase and redistribution of H3K27me3 can be caused by loss of the H3K9 methyltransferase complex 102. H3K36me3 is an important regulator of MMR pathway as earlier reported, and its imbalance may be due to the resultant interaction between damaged DNA and alterations in the level of epigenetic markers during senescence 103. The epigenetic inhibition of DNA damage repair genes caused by hypermethylation of MLH1 and MSH2 promoters, and the inefficient recruitment of MMR complex at DNA damage site caused by the decrease of H3K36me3 level can impair the MMR pathway 104. In addition, many studies have shown that histone deacetylases (HDACs) and histone acetyltransferases (HATs) play an important role in DNA repair and DNA damage response(DDR), by identifying DNA double strand breaks 105 and promoting non-homologous terminal connections 106. HDAC1 is also associated with other proteins involved in DDR, including proliferating cell nuclear antigens (PCNA), breast cancer 1 (BRCA1), ATM (ataxia telangiectasia mutated) and ATR (ATM and Rad3 related) 107. There is evidence that HDAC6 deacetylates and ubiquitinates MSH2, and regulates DDR and MMR activity. However, it is not known whether MSH2 is regulated by other HDAC (except HDAC6) and HATS 108.

Epigenetic alterations in histone modification represent a prominent marker of aging 109. Research on Drosophila found that aging leads to the loss of epigenetic marker distortion and the drift of H3K27me3, which in turn decrease expression of glycolysis genes, resulting in a negative effect on energy production and the redox state 110. The epigenetic regulation of chromatin remodeling on gene expression is crucial to the function of adult stem cells. The decline of stem cell function can be observed during aging, accompanied by currently unexplainable changes in chromatin structure 111. There is coordination between histone modifiers and chromatin remodeling resulting in a coordinated response to prevent DNA damage 112. Epigenetic proteins participate in DDR. “Active” chromatin modifiers such as HATS and chromatin remodeling complex are first recruited to the DNA damage site to enable DNA repair proteins to enter the “open” local chromatin structure. Once DNA repair proteins enter DNA, they recruit “inhibitory” chromatin modifiers such as HDAC to “turn off” chromatin and inhibit transcriptional activity. Finally, when the repair is completed, the chromatin returns to its original state 113. It has been proven that JAK2 is recruited into chromatin and JAK2 interacts with MSH2 and MSH6 in the nucleus. Inhibition of JAK2 changes the chromatin interaction between MSH2, MSH6, Dnmt1 and PRC2 members 114.

MicroRNAs (miRNAs or miRs), short (~22s nucleotide) single-stranded RNAs, act as gene expression regulators by binding to target mRNAs and disrupting their stability or inhibiting their translation 115-117. miRNAs have been shown to be involved in the DNA MMR pathway, a major genomic maintenance system. Evidence suggests that overexpression of miR-155 remarkably down-regulates core MMR proteins hMSH2, hMSH6, and hMLH1, inducing mutant phenotypes and MSI. The expression of miR-155 was found to be negatively correlated with the expression of MLH1 or MSH2 protein in human colorectal cancer 118. In mammalian cells, primary miRNA transcripts (pri-miRNAs) are initially processed into precursor miRNAs (pre-miRNAs) in the nucleus and further processed in the cytoplasm to produce mature miRNAs 115-117. *In vitro* and *in vivo* studies have demonstrated that MLH1 and miR-422a are involved in regulating feedback loops at both molecular levels. MutLα stimulated the transformation of pri-miR-422a into forward miR-422a, as well as the processing of other measured miRNAs, suggesting that MutLα is a general stimulator of miRNA biogenesis. In contrast, miR-422a down-regulated MutLα by pairing with MLH1 3'-untranslated region to inhibit MLH1 expression 119.

There is thus evidence that aging is related to genetic and epigenetic alterations. Given the reversibility of epigenetic mechanisms, its pathways provide a promising approach for the treatment of age-related decline and diseases 109.

## MMR and nutrient sensing

**Figure 5:**
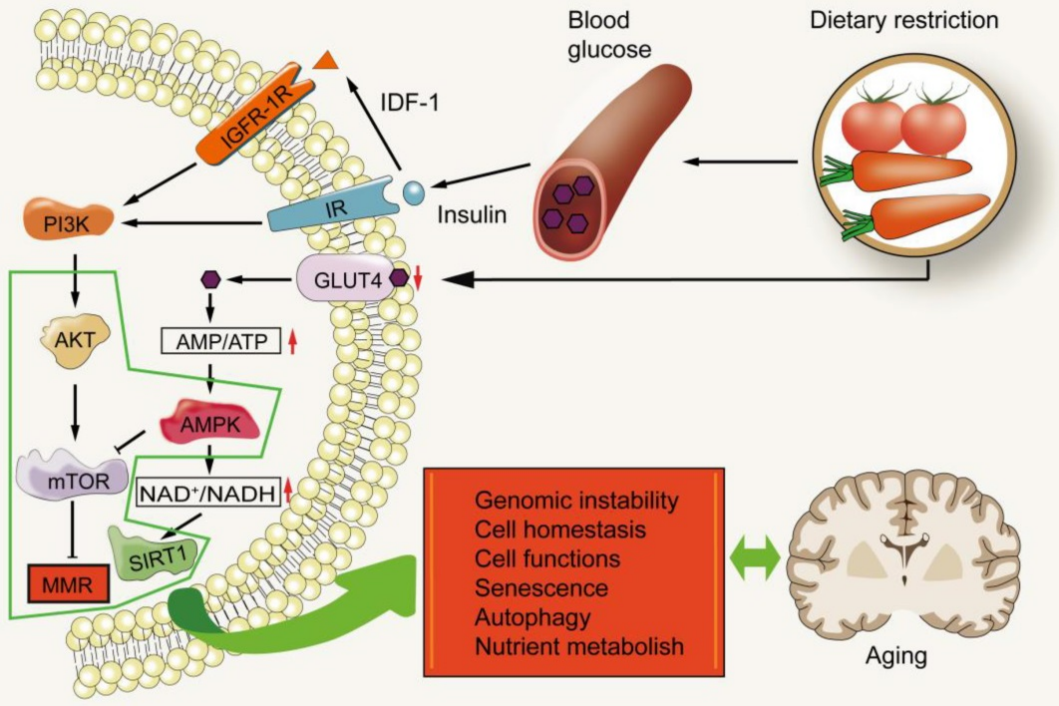
** MMR and nutrient sensing.** Dietary restriction leads to a decrease in blood glucose levels, which in turn lowers the levels of IGF-1 and insulin. This results in the decrease of downstream signal transduction of insulin receptor (IR) and insulin-like growth factor-1R (IGF-1R). During this time, mTOR is in an inactive state. The decrease in intracellular glucose utilization also increases the ratio of AMP/ATP, thus activating AMPK. AMPK can inhibit the ratio of mTOR complex 1. PI3K/AKT/mTOR may promote aging by inhibiting MMR.

Diet has been proven as a strong regulator of aging, and calorie restriction has become a valuable intervention (Figure 5), though many questions remain on how it affects aging-related processes. The regulation of nutrition sensing pathways diminishes with increasing of age, and eventually fails at advanced ages. These pathways form the link between diet and aging and can thus be regulated through drugs and dietary intervention. The nutrient sensing pathways of aging injury include IGF1/PI3K/AKT/mTOR and AMPK/Sirtuin/PGC1α. They medicate dominant roles in protein synthesis, cell cycle, DNA replication, autophagy, stress response and regulation of glucose homeostasis 120-123.

These pathways link metabolism, diet and aging. The growth axis of mammals includes growth hormone (GH) and insulin-like growth factor-1 (IGF-1). The intracellular signal pathway of IGF-1 is the same as that induced by insulin. Insulin informs cells of the presence of glucose 124, 125. High glucose levels induce insulin release, which in turn increases IGF-1. IGF-1 binds to its receptor, turns on its self-phosphorylation, and leads to the subsequent activation of phosphatidylinositol-3 kinase (PI3K) which in turn phosphorylates and activates AKT. Activated AKT phosphorylates and activates mTOR, to inhibit FOXO. Decreased glucose levels are detected by the insulin receptor which then diminishes this signal cascade reaction 121. mTOR, is a serine/threonine kinase, belonging to the phosphoinositide kinase related family. It can be found in two different complexes: mTOR complex 1 (MTORC1) and complex 2 (mTORC2). MTORC1 is activated by a variety of growth factors through the phosphatidylinositol-3 kinase-related family and the AKT kinase signal pathway. MTORC1 is also activated by nutrients (including amino acids and phosphates) and inhibited by AMP-activated protein kinase (AMPK). AMPK is the key sensor of cell energy state 126. In fact, low levels of insulin and IGF-1, two growth factors that activate mTOR, are induced by calorie restriction, and are associated with healthier lives and longer lifespans 127.

During starvation conditions, mTOR dissociates from ULK1-mAtg13-FIP200 complex and induces ULK1 to activate and phosphorylate mAtg13 and FIP200, thus promoting the translocation of the protein complex to an autophagy site 128. Mice subjected to intermittent fasting (IF) through dietary restrictions demonstrated better regulation of glucose homeostasis 129. Rapamycin was found to inhibit the activation of Akt, probably by relieving the negative feedback inhibition of mTOR-S6K1. Akt activated by rapamycin inhibiting mTOR may phosphorylate and inactivate its substrate, such as FOXO, which has recently been found to be involved in autophagy in Drosophila and mouse skeletal muscles 127. This research institution demonstrated that mTOR modulates the up-regulation of BNIP3 protein levels following MMR treatment with 6-TG and 5-FU 130. The two key targets of MTORC1 signal transduction are 4EBP1 and S6K, which are the regulatory factors of translation initiation. Inhibition of mTORC-dependent translation has been shown to prolong life and provide protection against several age-related diseases 131. The PI3K/AKT/mTOR pathway is often activated in human tumors and up-regulated in MMR-deficient tumors 132. Similarly, PI3K/AKT/mTOR pathway may also be up-regulated in senescent cells with MMR deficiency.

## MMR and cell senescence

**Figure 6:**
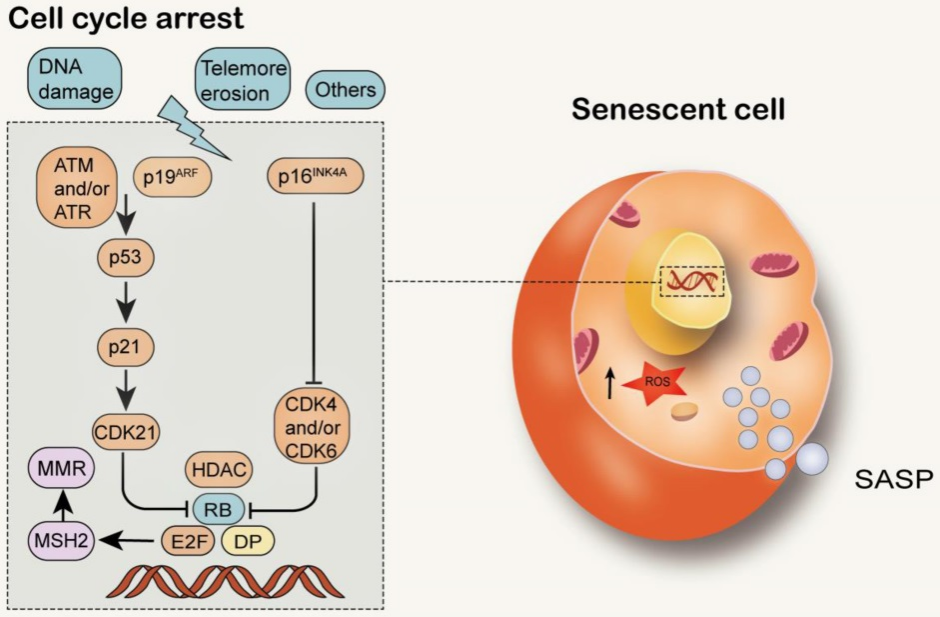
** MMR and cell senescence.** Structural DDR signal leads to chronic activation of p53, which leads to cell senescence. Cell cycle arrest during senescence is largely mediated by the activation of one or two of the p53/p21^WAF1/CIP1^ and p16^INK4A^/pRB tumor suppressor pathways. The decrease of E2F transcriptional activity in senescent cells can inhibit MSH2 and reduce the function of MMR.

Cell senescence is a permanent state of cell cycle stagnation, which promotes tissue remodeling during development and after injury, but may also lead to the decline of tissue regeneration potential and function, resulting in inflammatory tumorigenesis within elderly organisms 133. Senescent cells are alive with metabolic activity, yet have significant changes in gene expression and complex aging-related secretory phenotypes 134 (Figure 6). In young organisms, cell aging can prevent the proliferation of damaged cells, thereby protecting them from cancer and contributing to the dynamic balance of tissue. Therefore, aging may be a beneficial compensatory response 3. However, in older organisms, cell senescence may be harmful because it affects tissue repair and regeneration, and promotes tissue aging due to the accumulation of senescent cells, depletion of stem or progenitor cell compartments, and SASP secretion 135, 136. It has been found that senescent cells accumulate exponentially with age in a variety of tissues. Senescent cells are observed in atherosclerosis, diabetes, lung disease and many other age-related diseases 137-139. Cell senescence has both advantages and disadvantages to the health of organisms and is considered to be an example of evolutionary antagonism 140-144. The activation of P53/p21^WAF1/CIP1^ and p16^INK4A^/pRB tumor suppressor pathways plays a central role in regulating aging 145, 146. P53/p21^WAF1/CIP1^ is activated in response to DNA damage caused by telomere attrition, oxidative or carcinogenic stress 147, 148. When DDR is activated, ATM and ATR are phosphorylated, leading to the stabilization of p53 149. Enhanced p53 transcription factor activity increases the expression of CDKip21, which initially blocks the cell cycle 150. P16^INK4A^ inhibits the activities of CDK4 and CDK6, resulting in the decrease of Rb phosphorylation, S-phase entry and cell cycle arrest 151.

Classic replicative senescence involves cell cycle arrest caused by telomere shortening. Studies in several models have shown that the MMR system can regulate telomere maintenance, thereby changing cell senescence 152, as previously described in the “MMR and telomere attrition” section, above. Some experiments on young and senescent colon fibroblasts (CCD-18Co) and human embryonic lung fibroblasts (IMR90) showed that the activity of homologous MMR was significantly decreased in senescent cells, especially the down-regulated expression of MSH2 and MSH6 proteins. In addition, the activity of MMR could be restored by adding purified MutSα to the extract of senescent cells. It has been found that the decrease in E2F transcriptional activity in senescent cells is essential for the inhibition of MSH2. The expression of E2F1 in resting cells restored the expression of MSH2 and the activity of MMR, while the senescent cells lacking E2F1 could not restore the expression of MSH2 and the activity of MMR. These results suggest that the inhibition of E2F1 transcriptional activity in senescent cells leads to the stable inhibition of MSH2, which in turn induces the dysfunction of MutSα, and consequentially decrease of MMR ability 153. It has been confirmed that MSH2 participates in cell cycle arrest and apoptosis in different ways, depending on the degree of DNA damage 152. The maladjusted DNA repair pathway may make bone marrow mesenchymal stem cells prone to senescence or apoptosis, as well as reduce their proliferative and self-renewal characteristics. For example, DNA damage can impair telomere replication and activate DNA damage checkpoints to maintain mesenchymal stromal cells (MSCs) function^154^. The continuous down-regulation of DNA repair may play a role in the maintenance of aging phenotype, which is in turn related to the accumulation of irreparable DNA damage 155.

A growing body of evidence suggests that in addition to the accumulation of oxidative damage in cells, abnormal DNA repair may lead to cancer, brain disease, and premature aging 156. The BER and MMR pathways initiated by mismatched adenine and thymidine glycosylases (MutY/MUTYH and TDG or MBD4, respectively) identify and remove normal DNA bases from mismatched DNA double-stranded bodies 157, 158. In the DNA repair defective cell bacterial MutY, mammalian MMR and human TDG can act in an abnormal manner: MutY and TDG removed adenine and thymine that were opposite to the misincorporated 8-oxyguanine and damaged adenine, respectively, while MMR removed thymine that was opposite to O^6^-methylguanine 159, 160. Abnormal DNA repair pathways modified by oxidative bases are closely associated with age-related diseases. For example, abnormal BER and MMR pathways of guanine oxide residues lead to trinucleotide amplification, which is the basis of Huntington's disease, a severe inherited neurodegenerative syndrome 156.

## Conclusions and perspectives

Any type of mutation pattern in somatic cells is associated with genomic instability caused by exposure to DNA damage agents or failure to repair DNA, both of which can cause cancers due to increased mutation rates 25. The relationship between genomic instability and the characteristics of aging, and the development of age-related diseases has aroused interest in the DNA repair pathway as a potential anti-aging strategy. The MMR pathway plays an important role in identifying and repairing mismatch bases in the process of DNA replication and gene recombination in normal cells and cancer cells. Acquired MMR defects are found in most human cases of primary, secondary or recurrent hematological malignant tumors 161. In recent years, the combination of structural analysis and the latest real-time single molecule and cell imaging techniques have provided new and detailed insights into the thermally driven basis of the complete MMR process 82. Some clinical trials have shown that MMR defects or high microsatellite instability are significantly associated with long-term immunotherapy-related responses and improve prognosis of colorectal and non-colorectal malignant tumors treated with immune checkpoint inhibitors. Anti-programmed cell death-1 inhibitor pembrolizumab has been approved for MMR defect/microsatellite instability-highly refractory or metastatic solid tumors, and nivolumab has been approved for colorectal cancer patients with MMR defect/high microsatellite instability 162. Due to a biallele functional loss mutation in one of the MMR genes (PMS2, MSH6, MLH1, or MSH2), various degrees of antibody formation defects, ranging from IgA or selective IgG subclass defects to common variable immunodeficiency and high IgM syndromes, have been detected in a small number of patients with constitutional mismatch repair defect (CMMRD) 163. Currently, dMMR identification has two main clinically relevant areas—screening for inherited cancer syndromes, such as Lynch syndrome, and predicting responses to conventional chemotherapy and immunotherapy. In clinical practice, dMMR can be detected at the genetic, protein, or functional level 164. The relationship between DNA MMR protein defects and subsequent microsatellite instability has been widely studied, but the relationship between DNA MMR and aging needs to be further explored. This paper elucidates the direct and indirect relationships between DNA MMR system and telomere attrition, epigenetic alterations, mitochondrial dysfunction, nutrient sensing and cell senescence. In addition, the mechanism underlying its influence on aging is elucidated. MMR defects are associated with hematopoietic regeneration defects and stem cell depletion due to the accumulation of genomic instability 161. MSH2 is involved in cell cycle arrest and apoptosis through different pathways 165. After UV-B induced DNA damage, the MMR system promoted G2/M phase arrest 166. MSH3 accumulates in the cytoplasm due to its shuttle response to inflammation; reduced nucleoprotein MSH3 increases EMAST (elevated microsatellite alterations at selected tetranucleotide) and DNA damage 167. The MMR pathway is also of particular interest in neurodegenerative diseases because of its influence on somatic amplification of CAG repeats, which increase in length over time, especially in the brain 168. Somatic amplification of Huntington protein was associated with earlier onset of Huntington's disease 169 and more severe symptoms 170. In mouse models, amplification is blocked when MSH2 and MSH3, MLH1 or PMS2 171-174. This evidence suggests that the MMR pathway is involved in disease progression 175. MMR may also interact with protein imbalance, stem cell failure, and information exchange between cells, thus promoting aging. MMR systems may be a reliable therapeutic target, opening up new treatments for neurodegenerative diseases, and most importantly, may help to control the progression of aging-related diseases.
